# Impact of COVID-19 containment measures on patients with rheumatic and musculoskeletal disease in the UK and Europe: the REUMAVID study (phase1)

**DOI:** 10.1093/rap/rkab098

**Published:** 2021-12-04

**Authors:** Stephanie Rose Harrison, Marco Garrido-Cumbrera, Victoria Navarro-Compán, José Correa-Fernández, Dale Webb, Laura Christen, Helena Marzo-Ortega

**Affiliations:** 1 National Institute of Health Research (NIHR) Leeds Biomedical Research Centre, Leeds Teaching Hospitals Trust; 2 Leeds Institute of Rheumatic and Musculoskeletal Medicine (LIRMM), University of Leeds, Leeds, UK; 3 Health and Territory Research, Universidad de Sevilla, Seville, Spain; 4 Axial Spondyloarthritis International Federation (ASIF), London, UK; 5 Hospital Universitario La Paz, IdiPAZ, Madrid, Spain; 6 National Ankylosing Spondylitis Society (NASS), London, UK; 7 Patient Engagement, Novartis Pharma AG, Basel, Switzerland

**Keywords:** rheumatic and musculoskeletal diseases, COVID-19, pandemic, well-being, mental health, United Kingdom, Europe

## Abstract

**Objectives:**

The aim was to compare the impact of the first wave of the coronavirus disease 2019 (COVID-19) pandemic and lockdown measures on patients with rheumatic and musculoskeletal diseases (RMDs) in the UK and other European countries (OEC).

**Methods:**

REUMAVID was an online cross-sectional survey of seven European countries. The data collected included the following: demographics, lifestyle, employment, access to health-care services, disease-specific characteristics, the World Health Organization five well-being index (WHO-5), hospital anxiety and depression scale (HADS), visual analogue scale (VAS) disease activity, and the perceived acceptable symptom scale.

**Results:**

One thousand eight hundred responses were received between April and July 2020 [UK, *n* = 558 (31.0%); OEC, *n* = 1242 (69.0%)]. UK patients were more likely to be older [mean (S.d.): UK 58.5 (13.4) years; OEC 50.0 (12.2) years], university educated [UK *n* = 302 (54.1%); OEC *n* = 572 (46.1%), quit smoking [UK *n* = 92 (59.4%); OEC *n* = 65 (16.2%)] and continue exercise [UK, *n* = 216 (49.2%); OEC, *n* = 228 (33.1%)], although, conversely, alcohol consumption increased [UK *n* = 99 (36.3%); OEC *n* = 98 (12.1%)]. UK patients felt informed about COVID-19 (UK 72.7%, OEC 57.4%) and kept their planned rheumatology [UK *n* = 87 (51.2%); OEC *n* = 213 (38.6%)] and/or general practice appointments [UK *n* = 87 (76.3%); OEC *n* = 310 (53.9%)]. Almost half the patients with RMDs reported a decline in health and well-being, although this was less common in UK patients [UK *n* = 214 (38.4%), OEC *n* = 618 (50.2%)], who reported better perceived acceptable symptom scale, VAS pain and HADS scores, but worse WHO-5 scores.

**Conclusions:**

UK RMD patients performed better in the physical and mental health domains tested, possibly owing to a less restrictive lockdown and better health-care access. These findings have implications for health-care services globally in planning patient care after the COVID-19 pandemic.

Key messages

- The coronavirus disease 2019 pandemic negatively affected the physical and mental health of European rheumatic and musculoskeletal disease patients.
- Compared with other European counterparts, UK patients performed better in nearly all health domains tested.
- The insights from this survey could help to shape the care of rheumatic and musculoskeletal disease patients post-pandemic.

## Introduction

The COVID-19 pandemic has, and continues to, put an unprecedented strain on health-care systems globally. The speed at which both community and hospital health-care services reorganized to accommodate the influx in COVID-19 patients was remarkable; however, non-COVID-19 acute and elective services suffered significantly as a result [1, 2]. Patients with rheumatic and musculoskeletal diseases (RMDs) are potentially more likely to be adversely affected owing to a combination of factors, including reduced detection of flare or adverse treatment effects, lack of continuity of care and access to specialist support, and the requirement for stricter social distancing and shielding practices for those taking immunosuppressive medications [3–6]. In addition, patients with RMDs are known to have associated morbidities and poorer baseline physical and mental well-being when compared with otherwise healthy individuals [7–9].

Strategies to manage the impact of the pandemic locally, regionally, nationally and internationally have varied vastly, reflecting both rapidly evolving research and understanding of severe acute respiratory syndrom coronavirus 2 (SARS-CoV-2) and the inherent differences between populations, health-care systems and the wider socio-economic context [10]. Patients and clinicians alike faced an insurmountable task in interpreting conflicting information from the media, health-care organizations, charities and governments [11], with individual nations adopting different containment measures; for example, outdoor activities and exercise were permitted in the UK, whereas in Spain and Italy people were largely confined indoors [10, 12]. Crucially, although stricter lockdown measures might provide more significant reassurance of protection from the virus, the detrimental effects on general health and well-being are also potentially greater. A better understanding of the impact of the pandemic on patients with RMDs is now needed to help rheumatologists and health-care systems address its short- and long-term health effects [13].

The REUMAVID study aimed to assess the impact of the COVID-19 pandemic in people with rheumatic and musculoskeletal disorders across European countries. In the present study, we compared the impact of the first wave of the COVID-19 pandemic and associated lockdown measures on rheumatology patients in the United Kingdom (UK) with RMD patients in other European countries (OEC).

## Methods

REUMAVID is a multidisciplinary international consortium assembled at the start of the pandemic with the aim of assessing its impact on the physical/mental health and overall well-being of patients with RMDs from participating European countries (Cyprus, France, Greece, Italy, Portugal, Spain and the UK), as described in detail previously [14]. Patients were recruited through national RMD patient groups that disseminated the survey link via their organizational websites. All participants were aged ≥18 years, residing in a REUMAVID-participating country, who had or were expected to have an appointment with their rheumatologist within 12 months of study entry. Before completing the survey, all patients provided informed consent electronically via the online survey platform. REUMAVID was first approved by the ethical committee of University Hospital La Paz under the code PI-4121 and subsequently approved in all other participating countries as legally required.

Patients first self-reported their diagnosis from the following list: ankylosing spondylitis/axial spondyloarthritis, FM, gout, JIA, myositis (PM or DM), OA, osteoporosis, peripheral spondyloarthritis, PMR, PsA, RA, synovitis, acne pustulosis hyperostosis and osteitis (SAPHO), SS, SLE, SSc (or scleroderma), vasculitis or arteritis.

They then completed a 120-question survey covering multiple domains of physical and mental health and well-being including demographics, lifestyle habits and weight, provision of information on COVID-19, measures of disease activity and mental/physical health, and four validated scales to measure well-being: the World Health Organization five well-being index (WHO-5), which measures overall well-being [15]; the hospital anxiety and depression scale (HADS) to measure anxiety/depression levels [16, 17]; a visual analogue scale (VAS) for disease activity; and the patient acceptable symptom scale [18].

Survey data were analysed using SPSS v.25.0 (IBM Corp., Armonk, NY, USA). For continuous parametric variables, the mean (S.d.) is reported. Statistical significance between groups (the UK and OEC) were determined using Mann-Whitney test for individual variables. Categorical outcome variables are reported as a frequency/percentages, and statistically significant differences between the UK and OEC were determined using the χ^2^ test. Sample size is reported for each individual variable to highlight any areas of missing data.

## Results

Overall, 2731 patients with RMDs participated in the first phase of the study. Owing to those patients who completed <70% of the survey, data for 931 patients had to be discarded, leaving 1800 patients for data analysis, of whom 558 respondents (31%) were recruited by the National Ankylosing Spondylitis Society, the National Rheumatoid Arthritis Society and Arthritis Action in the UK.

### Demographics

Compared with OEC, UK participants were older [UK 58.5 (13.4) years; OEC 50.0 (12.2) years, *P* < 0.001]. Overall, the vast majority of respondents were female, both in the UK and in the OEC [UK 78.7% (439), OEC 81.0% (1003), *P* = 0.262] and identified themselves as being married or in a relationship [UK 70.8% (395), OEC 69.1% (858), *P* = 0.008], with similar proportions of respondents from both the UK and OEC being members of patient organizations [UK 42.4% (236), OEC 41.2% (512), *P* = 0.627; Table 1]. In contrast, UK participants were more likely to have obtained a university qualification [UK 54.1% (302), OEC 46.1% (572), *P* < 0.001] and to be retired [UK 35.5% (198), OEC 16.5% (205), *P* < 0.001; Table 1]. Interestingly, among working participants teleworking was more common in the UK [UK 58.1% (144), OEC 32.9% (215), *P* < 0.001; Table 1].

**Table 1:** Socio-demographic, working life and anthropometric characteristics, lifestyle habits and outdoors contact during the coronavirus disease 2019 pandemic

Characteristic	Mean (S.d.) or *n* (%)		*P*-value
	UK 558 (31.0%)	OEC 1242 (69.0%)	
Age, years	58.5 (13.4)	50.0 (12.2)	<0.001^*^
Gender (female), *n* = 1797	439 (78.7)	1003 (81.0)	0.262
Marital status			
Single	81 (14.5)	206 (16.6)	0.008*
Married/in relationship	395 (70.8)	858 (69.1)	
Separated/divorced	51 (9.1)	144 (11.6)	
Widower/widow	31 (5.6)	34 (2.7)	
Educational level			
No schooling completed	13 (2.3)	7 (0.6)	<0.001*
Primary school	0 (0.0)	72 (5.8)	
Secondary school	94 (16.8)	213 (17.1)	
Vocational qualification	149 (26.7)	378 (30.4)	
University	302 (54.1)	572 (46.1)	
Employment status			
Retired	198 (35.5)	205 (16.5)	<0.001*
Teleworking, *n* = 901	144 (58.1)	215 (32.9)	<0.001*
Patient organization (member), *n* = 1798	236 (42.4)	512 (41.2)	0.627
BMI, kg/m^2^, *n* = 1513			
Underweight (<18.5)	14 (4.2)	41 (3.5)	0.011*
Normal weight (18.5–24.9)	127 (38.4)	501 (42.4)	
Overweight (25–29.9)	86 (26.0)	368 (31.1)	
Obesity (>30)	104 (31.4)	272 (23.0)	
Gaining weight during COVID-19 pandemic (yes)	219 (39.2)	517 (41.6)	0.561
Smoking during COVID-19 pandemic, *n* = 556			
More than before	16 (10.3)	121 (30.2)	<0.001*
Same as before	34 (21.9)	153 (38.2)	
Less than before	11 (7.1)	46 (11.5)	
I’ve started smoking	2 (1.3)	16 (4.0)	
I’ve quit smoking	92 (59.4)	65 (16.2)	
Alcohol consumption during COVID-19 pandemic, *n* = 1085			
More than before	99 (36.3)	98 (12.1)	<0.001*
Same as before	137 (50.2)	267 (32.9)	
Less than before	36 (13.2)	100 (12.3)	
I’m not drinking	1 (0.4)	347 (42.7)	
Physical activity during COVID-19 pandemic, *n* = 1128			
Yes	216 (49.2)	228 (33.1)	<0.001*
No	91 (20.7)	423 (61.4)	
No, but it was compensated by other exercise	132 (30.1)	38 (5.5)	
Outdoors contact during COVID-19 pandemic			
Visits to natural environment (yes), *n* = 1429	288 (51.9)	275 (31.5)	<0.001*
Walking outside (every day)	214 (38.4)	276 (22.2)	<0.001*
Groceries shopping, *n* = 591			
Going as usual	34 (22.1)	175 (40.0)	<0.001*
Someone from my household	44 (28.6)	156 (35.7)	
Someone from outside my household	11 (7.1)	28 (6.4)	
Online or by phone	65 (42.2)	78 (17.8)	

*n* = 1800, unless otherwise specified. *Statistically significant at *P* < 0.05. COVID-19: coronavirus disease 2019; OEC: other European countries; UK: United Kingdom.

### Weight and lifestyle

More than half of the study participants were overweight or obese [BMI >25 kg/m^2^; UK 57.4% (190), OEC 54.1% (640)], with rates of obesity (BMI >30 kg/m^2^) being higher in the UK population [UK 31.4% (104), OEC 23.0% (272), *P* = 0.011; Table 1]. In addition, 39.2% (219) of the UK participants and 41.6% (517) in the OEC reported gaining weight during the first-wave period (*P* = 0.561). Almost one-third of participants reported smoking (31%: UK = 155, OEC = 401), although in the UK, 59.4% (92) of participants said they had quit smoking, compared with 16.2% (65) in OEC (*P* < 0.001; Table 1). The converse was true for alcohol consumption, with 60.3% of all respondents reporting alcohol consumption and with UK participants admitting to drinking more [UK 36.3% (99), OEC 12.1% (98), *P* < 0.001] or the same amount [UK 50.2% (137), OEC 32.9% (267), *P* < 0.001] compared with their European counterparts, whereas nearly half of the respondents in the OEC did not drink at all [UK 0.4% (1), OEC 42.7% (347), *P* < 0.001; Table 1]. UK participants were more likely to have continued physical activity during the pandemic [UK 49.2% (216), OEC 33.1% (228), *P* < 0.001], to visit a natural environment [UK 51.9% (288), OEC 31.5% (275), *P* < 0.001] and/or to go for daily walks outside [UK 38.4% (214), OEC 22.2% (276), *P* < 0.001]. They were also more likely to do grocery shopping online [42.4% (65), OEC 17.8% (78), *P* < 0.001], whereas in OEC, patients reported that someone from their household would go to the shop in person (*P* < 0.001; Table 1).

### Access to rheumatology services

More than half of the rheumatology patients in the UK were able to keep scheduled rheumatology appointments during the pandemic, which was more than in the OEC (UK 51.2 *vs* 38.6%, *P* = 0.004; Table 2). They were also more likely to be able to discuss the effects of treatment on COVID-19 with either their rheumatologist (81.0 *vs* 57.6%, *P* < 0.001) or general practitioner (76.3 *vs* 53.9%, *P* < 0.001). In contrast, around half of the patients reported not being able to continue their psychological/psychiatric therapies either online or via telephone, with no significant difference in the UK compared with OEC (45.7 *vs* 52.2%, *P* = 0.459; Table 2).

**Table 2:** Health-care utilization and psychological or psychiatric care during coronavirus disease 2019 pandemic

Health care	Mean (S.d.) or *n* (%)		*P*-value
	UK 558 (31.0%)	OEC 1242 (69.0%)	
Health-care utilization			
Scheduled appointment with rheumatologist, *n* = 722			
Yes	87 (51.2)	213 (38.6)	0.004*
Contact with rheumatologist about possible effects of treatment on COVID-19, *n* = 430			
Yes	94 (81.0)	181 (57.6)	<0.001*
Access to primary care or general practitioner, *n* = 689			
Yes	87 (76.3)	310 (53.9)	<0.001*
Psychological or psychiatric care			
Not continuing psychological/psychiatric therapy (online or by telephone), *n* = 437	16 (45.7)	210 (52.2)	0.459

*n* = 1800, unless otherwise specified. *Statistically significant at *P* < 0.05. COVID-19: coronavirus disease 2019; OEC: other European countries; UK: United Kingdom; VAS: visual analogue scale.

### Access to information on COVID-19

**Fig. 1:**
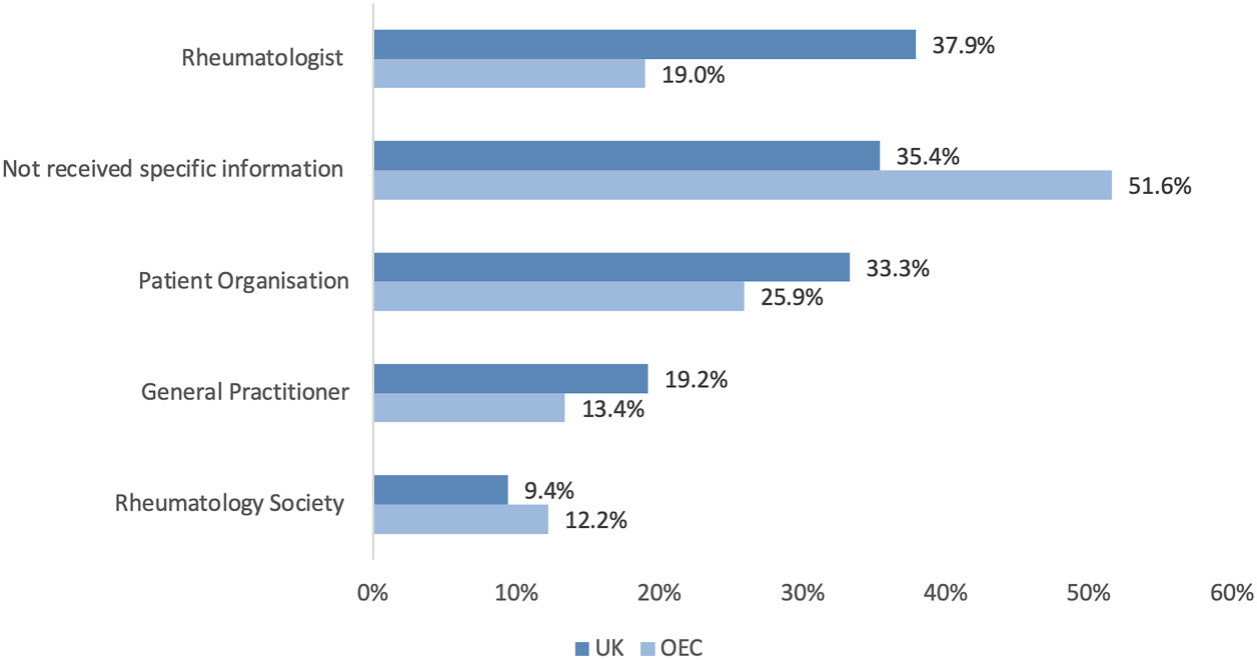
Information received about how coronavirus disease 2019 might affect your rheumatic disease The information received about how COVID-19 might affect their condition was better rated by the UK sample (3.2 out of 5) *vs* the OEC (2.8 out of 5), and this difference was statistically significant (*P* < 0.001). *n* = 1682. COVID-19: coronavirus disease 2019; OEC: other European countries; UK: United Kingdom.

Overall, 35.4% of patients in the UK reported receiving no information on how COVID-19 might affect their RMD, compared with 51.6% in the OEC (*P* < 0.001; Fig. 1). Rheumatologists were the main source of information for patients on how COVID-19 might affect their RMD, with twice as many UK patients (37.9%) reporting receiving such information *vs* those in the OEC (19.0%) (Fig. 1). The next most common source for this information was patient organizations (UK 33.3%, OEC 25.9%), followed by general practitioners (UK 19.2%, OEC 13.4%) and the national rheumatology societies (UK 9.4%, OEC 12.2%; Fig. 1).

**Fig. 2:**
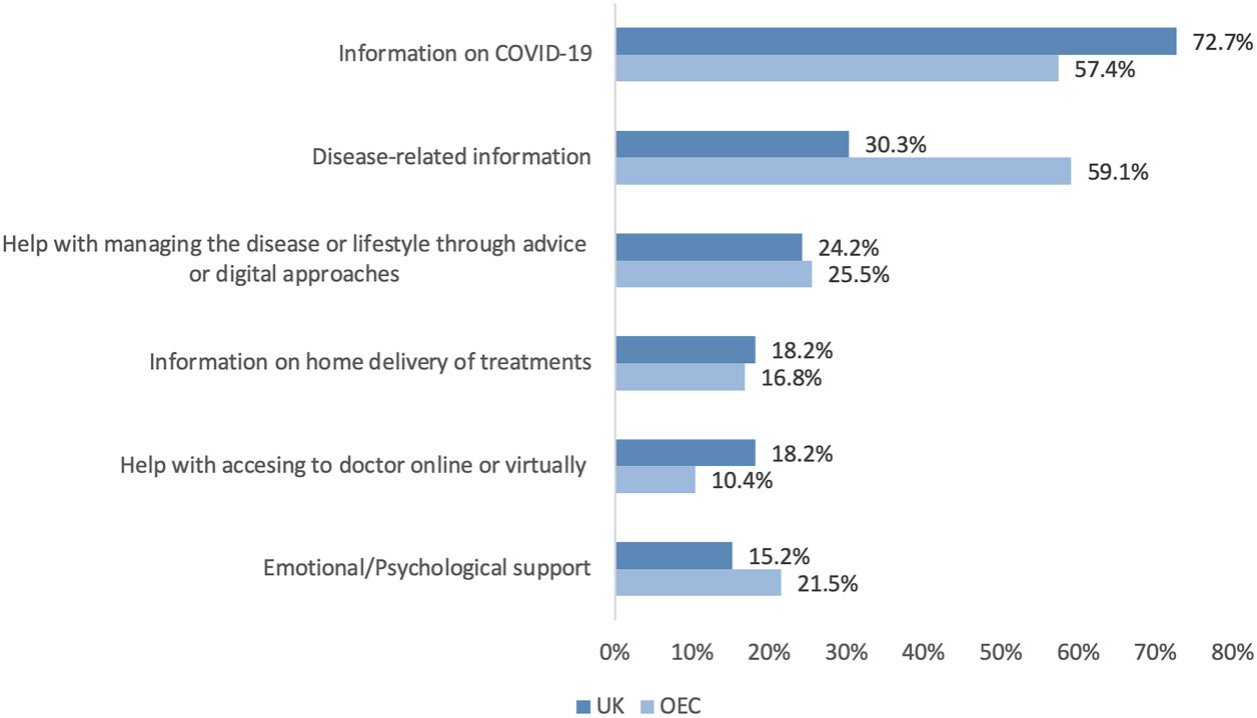
Type of support of patients from a patient organization OEC: other European countries; UK: United Kingdom. *n* = 331

Interestingly, although patient organizations played an important role in providing information for patients in all countries, the focus of the information differed. For instance, in the UK, patient organizations provided more COVID-19-related information (UK 72.7%, OEC 57.4%), whereas in the OEC the patient groups provided more disease-related information (UK 30.3%, OEC 59.1%, *P* = 0.017; Fig. 2). Less information was provided overall on how to access their rheumatologist, home delivery of treatments and emotional/psychological support in both the UK and OEC (Fig. 2).

### Disease-specific characteristics and measures of overall health/well-being

In the UK, 54.2% (302) of patients reported good or very good self-perceived health, compared with 27.8% (342) in OEC (*P* < 0.001), and UK patients were less likely to report a deterioration in their health, with 38.4% (214) in the UK reporting worsening of their health compared with 50.2% (618) in the OEC (*P* < 0.010; Table 3). More than two-thirds of patients in the UK and OEC reported increased disease activity, which was slightly less in the UK cohort [UK 67.6% (374), OEC 76.5% (920), *P* < 0.001; Table 3). Levels of anxiety and depression were also worse in the OEC [HADS anxiety: UK 43.6% (241), OEC 63.5% (772), *P* < 0.001; HADS depression: UK 33.6% (186), OEC 51.4% (625), *P* < 0.001], although poorer overall well-being was reported by UK patients [WHO-5 ≤ 50: UK 52.5% (292), OEC 47.3% (578), *P* = 0.043; Table 3].

**Table 3:** Disease-specific characteristics

Characteristic	Mean (S.d.) or *n* (%)		*P*-value
	UK 558 (31.0%)	OEC 1242 (69.0%)	
Self-perceived health			
Very good	66 (11.8)	59 (4.8)	<0.001*
Good	236 (42.4)	283 (23.0)	
Fair	207 (37.2)	595 (48.4)	
Bad	47 (8.4)	246 (20.0)	
Very bad	1 (0.2)	46 (3.7)	
Change in health status during lockdown			
Much worse than before	23 (4.1)	159 (12.9)	<0.001*
Moderately worse	191 (34.3)	459 (37.3)	
Same as before	305 (54.8)	538 (43.8)	
Moderately better	34 (6.1)	63 (5.1)	
Much better than before	4 (0.7)	10 (0.8)	
VAS disease activity (≥4), *n* = 1756	374 (67.6)	920 (76.5)	<0.001*
VAS pain (≥4), *n* = 1421	373 (67.7)	701 (80.6)	<0.001*
WHO**-**5, *n* = 1777			
Poor wellbeing (WHO-5 ≤ 50)	292 (52.5)	578 (47.3)	0.043*
HADS anxiety (0–21), *n* = 1769			
Risk (8–21)	241 (43.6)	772 (63.5)	<0.001*
HADS depression (0–21), *n* = 1769			
Risk (8–21)	186 (33.6)	625 (51.4)	<0.001*

*n* = 1786 unless otherwise specified. *Statistically significant at *P* < 0.05. HADS: hospital anxiety and depression scale; OEC: other European countries; UK: United Kingdom; VAS: visual analogue scale; WHO-5: the World Health Organization five well-being index.

## Discussion

As the focus shifts from immediate treatment of SARS-CoV-2-infected patients and towards mitigating the effects of the pandemic on patients with chronic health conditions, there is a significant need to gain a better understanding of the indirect effects of the ongoing COVID-19 pandemic on the overall well-being and disease activity of patients with RMDs. This survey compared and contrasted the experience of UK patients with that of OEC patients during the first wave of the COVID-19 pandemic. We felt that this analysis was of interest because political decisions led to different approaches in containment and lockdown measures throughout Europe. British respondents were generally older, with higher educational attainment and more likely to be retired, which is similar to previous surveys [19, 20]. As expected from the less stringent containment measures in the UK, more British patients continued to exercise, often outdoors or in a natural environment. They also reported quitting smoking, all of which have positive impacts on physical and mental health, although conversely, many UK respondents increased their alcohol consumption. Teleworking and online/telephone grocery shopping were also more common in the UK. British patients were more likely to keep their appointment with their rheumatologist or general practitioner, received more information on COVID-19 and had higher levels of satisfaction with the quality of that information. Self-reported health status, anxiety and depression levels were better in UK patients, although overall levels of well-being were comparatively worse, possibly owing to the self-reported nature of the instrument used [21].

Despite the comparatively better outcomes for British patients, there was still a significant burden associated with lockdown measures. One reason for this was the significant challenges that all health-care organizations faced in assimilating and disseminating high-quality guidelines in a rapidly changing research environment with no clear standard of best practice. At the start of the pandemic, EULAR published principles for the care of patients with RMDs; however, there were significant ramifications to their recommendations and many obstacles to their implementation, with lockdown, redeployment of health-care staff and strict social distancing [22]. As a result, less than half of the patients kept their rheumatology appointment (UK 51.2%, OEC 38.6%, *P* = 0.004), the impact of which is reflected in the self-reported disease-specific characteristics seen in this survey.

Nevertheless, British patients still fared better overall, which might be attributable, in part, to less stringent government restrictions. Researchers at the University of Oxford developed a scoring system to compare the stringency of lockdown measures between countries quantitatively [10]. Using this resource, between January and June 2020, the UK had the least strict measures compared with the OEC. Given that adherence to lockdown rules in the first wave was high, especially among patients with RMDs who were considered vulnerable/extremely vulnerable [23, 24], it seems reasonable to assume that the stringency of lockdown measures would impact on overall health and well-being.

One notable difference between lockdown measures was that British patients were more likely to continue to exercise outdoors. Exercise and outdoor activity reduce anxiety and depression, improve overall well-being [25, 26], maintain mobility and reduce pain in patients with RMDs [5]. British patients also felt that they received better-quality RMD-specific guidance on COVID-19, which is likely to have provided some reassurance at a time of great uncertainty. In addition, more UK patients reported already belonging to patient organizations before the pandemic, and it is possible that having these support networks already established helped British patients to feel more connected to others in their position and facilitated rapid access information, making them feel empowered. Furthermore, UK patients appeared to use more digital technologies; for example, in grocery shopping online and teleworking, which might have provided a routine and some semblance of normality, contributing to improved resilience during the first wave.

This study is the first to report the impact of the first wave of the COVID-19 pandemic on the physical/mental health and overall well-being of patients from seven participating European countries, providing valuable insight into the impact associated with the different containment measures initiated in each country. These results can inform the future planning and delivery of services for patients with RMDs in Europe. There are some limitations, however, similar to other survey studies, with an over-representation of females, those who are retired and those with higher levels of educational attainment [27]. Nevertheless, the sex distribution might be related to the distribution of rheumatic disease in the survey, although approximately one-third of respondents had axial spondyloarthritis, which is more frequently diagnosed in males. Those with higher educational attainment are also more likely to engage with patient support groups and self-help measures and to be able easily to access and understand the rapidly evolving information on the pandemic. Conversely, those who work might have been deterred from participation in the survey owing to the length of time it would take to complete 120 questions, and those from lower socio-economic background and/or ethnic minority groups might not have access to adequate digital facilities or be able to access the survey in their native language [28]. Therefore, the relative impact of the pandemic on those of working age, males and those from a deprived background or ethnic minority group is unclear. In addition, the distribution of RMDs in this survey does not reflect the prevalence of RMDs in real-world practice, with over-representation of axial spondyloarthritis and RA, reflecting the instrumental role played by the patient societies collaborating in the REUMAVID study.

Furthermore, the patient survey does not allow us to detect inter-regional and local differences in the patient experience, which is key information for care providers who need to use already stretched local resources effectively in the aftermath of the first wave of the pandemic. This is even more true of the second wave of the pandemic in the UK, where inter-regional lockdown practices differed significantly compared with the first wave. Future surveys should therefore seek to target under-represented groups and provide local and regional data. This survey is cross-sectional and, as alluded to above, experiences might well differ between different phases of the pandemic, even within the same country. Likewise, our survey does not include all European countries, and the patient experiences there, or indeed in non-European nations, could differ substantially. Finally, there is no control group of patients completing the survey before the pandemic, although in our survey, patients, when asked, did report that their health had worsened relative to the pre-pandemic state.

In conclusion, these data suggest that UK patients with RMDs performed better than OEC patients in the physical and mental health domains tested. UK respondents reported less smoking and greater levels of physical activity, although they consumed more alcohol, and they showed greater adaptation to digital platforms with increased use of online shopping and teleworking. These differences might be attributable to a multitude of factors, both known and unknown. Nevertheless, the REUMAVID survey does highlight some factors that could have played a role in the observed differences, including the fact that UK patients were living under comparatively less stringent lockdown measures with better access to outdoors spaces, health-care professionals and advice on COVID-19. Irrespective of the reasons underlying the differences, the REUMAVID survey highlights the clear negative effects of the first wave of the pandemic on the mental health of all patients with RMDs, even if UK respondents reported comparatively less anxiety and depression. These findings have broad implications for health-care services globally in planning patient care in the aftermath of the pandemic.
